# Structural Consequence of Non-Synonymous Single-Nucleotide Variants in the N-Terminal Domain of LIS1

**DOI:** 10.3390/ijms23063109

**Published:** 2022-03-14

**Authors:** Ho Jin Choi, Sarmistha Mitra, Yeasmin Akter Munni, Raju Dash, Sarmin Ummey Habiba, Md Sohel, Sultana Israt Jahan, Tae Jung Jang, Il Soo Moon

**Affiliations:** 1 Department of Anatomy, Dongguk University College of Medicine, Gyeongju 38066, Korea; chjack@naver.com (H.J.C.); sarmisthacu@gmail.com (S.M.); yeasminakteracce@gmail.com (Y.A.M.); rajudash.bgctub@gmail.com (R.D.); 2 Department of Pharmacy, BGC Trust University Bangladesh, Chittagong 4381, Bangladesh; sarmin.ummey.habiba07@gmail.com; 3 Department of Biochemistry and Molecular Biology, Mawlana Bhashani Science and Technology University, Santosh, Tangail 1902, Bangladesh; mdsohel3921@gmail.com; 4 Department of Biotechnology and Genetic Engineering, Noakhali Science and Technology University, Noakhali 3814, Bangladesh; sultanajahan75@gmail.com; 5 Department of Pathology, Dongguk University College of Medicine, Gyeongju 38066, Korea; taejung@mail.dongguk.ac.kr

**Keywords:** *LIS1*, single nucleotide polymorphisms, molecular dynamics simulation, variant, lissencephaly

## Abstract

Disruptive neuronal migration during early brain development causes severe brain malformation. Characterized by mislocalization of cortical neurons, this condition is a result of the loss of function of migration regulating genes. One known neuronal migration disorder is lissencephaly (LIS), which is caused by deletions or mutations of the *LIS1* (PAFAH1B1) gene that has been implicated in regulating the microtubule motor protein cytoplasmic dynein. Although this class of diseases has recently received considerable attention, the roles of non-synonymous polymorphisms (nsSNPs) in *LIS1* on lissencephaly progression remain elusive. Therefore, the present study employed combined bioinformatics and molecular modeling approach to identify potential damaging nsSNPs in the *LIS1* gene and provide atomic insight into their roles in LIS1 loss of function. Using this approach, we identified three high-risk nsSNPs, including rs121434486 (F31S), rs587784254 (W55R), and rs757993270 (W55L) in the *LIS1* gene, which are located on the N-terminal domain of LIS1. Molecular dynamics simulation highlighted that all variants decreased helical conformation, increased the intermonomeric distance, and thus disrupted intermonomeric contacts in the LIS1 dimer. Furthermore, the presence of variants also caused a loss of positive electrostatic potential and reduced dimer binding potential. Since self-dimerization is an essential aspect of LIS1 to recruit interacting partners, thus these variants are associated with the loss of LIS1 functions. As a corollary, these findings may further provide critical insights on the roles of LIS1 variants in brain malformation.

## 1. Introduction

The lissencephaly 1 gene, *LIS1 (PAFAH1B1)*, located in chromosome 17p13.3, is one of the major genes associated with neural migration [1,2], a fundamental process of cortex development where postmitotic neurons migrate to their appropriate positions to ensure proper spatial relationships with other cells [3]. The genomic region of *LIS1* contains 11 exons, and 10 of that encode the protein of 150 kDa [4], which is highly conserved and differs only one and three amino acids between humans, mice, and cattle, respectively, and showed 70% similarity with drosophila [5,6,7].

LIS1 has been linked to different stages of neuronal development, especially in neuronal migration and neurite outgrowth [8]. Functional information about the *LIS1* gene was first disclosed in *Aspergillus nidulans* regulating microtubules formation and microtubules-based motors [9]. Sapir et al. found that LIS1 interacts with tubulin and modulates microtubule dynamics, indicating a conserved evolutionary function of the *LIS1* gene [10]. Interestingly, overexpression of *LIS1* in non-neuronal cells increased retrograde movement of cytoplasmic dynein and promoted the peripheral accumulation of microtubules, reflecting the acquisition of neuron-like dynein behaviors [11]. LIS1 interacts with numerous proteins, including dynein (DYNC1H1) [6], NDEL1 [12], CLIP1 [13], NUDC [14], TUBA1A [15], NAGK [16,17], and doublecortin [18], and thus promotes essential cellular functions such as cellular transport, proliferation, and migration [19].

Accumulating studies suggested that deficiency or mutation of the *LIS1* gene attributes the opposite effect, responsible for a developmental disorder called lissencephaly, manifesting typical facial dysmorphology with more severe brain malformation [19,20,21]. *LIS1* mutations are pronounced in about 60–75% of patients with isolated lissencephaly sequence (ILS) [22,23], where the malformations caused by LIS1 mutation result in severe developmental delays that most patients do not reach measurable developmental stages. *LIS1* knock-out mice display disorganization of cortical, hippocampal, and olfactory organs, delay neural migration [23], and impair retrograde organelles transport in neuronal cells [24]. Up to 2003, only five missense mutations have been found in patients [25]. Sporadic mutation of the *LIS1* gene causes Type I lissencephaly (1), resulting in haploinsufficiency 2 [26].

Single-nucleotide polymorphism (SNP) is a common type of nucleotide modification or genetic variant in a single genetic code [27], occurring in every 200–300 base pair [28] with a high frequency in both noncoding and coding regions. Amino acid change in coding regions (about 0.5 million SNPs in humans) [29], which is caused by non-synonymous SNP (nsSNP), can impact phenotypic characteristics, including structure, charge, stability, solubility, and functions of the protein [30,31,32]. However, it is sometimes impossible to understand the disease-causing mutations in complex diseases using an experimental approach due to its time-consuming and expensive experiments and thus often overlooked in research [33,34]. In contrast to experimental approaches, in silico techniques that can analyze the roles of nsSNPs can substitute experimental approaches to investigate their impact on protein structure, stability, and function. For instance, several in silico studies have identified the effects of deleterious nsSNPs on the structure and function of the various protein [34,35,36]. To date, there is a lack of intensive nsSNPs study of the *LIS1* gene, and it is expected that there is exist nsSNP in *LIS1* gene that has a considerable impact on its functions. Therefore, we aimed to investigate the significant roles of the targeted gene-coded protein with the structural and functional consequence of nsSNPs of LIS1 protein using a wide range of bioinformatics tools and molecular dynamics (MD) simulation.

## 2. Results

### 2.1. Screening of Most Deleterious nsSNPs

**Figure 1:**
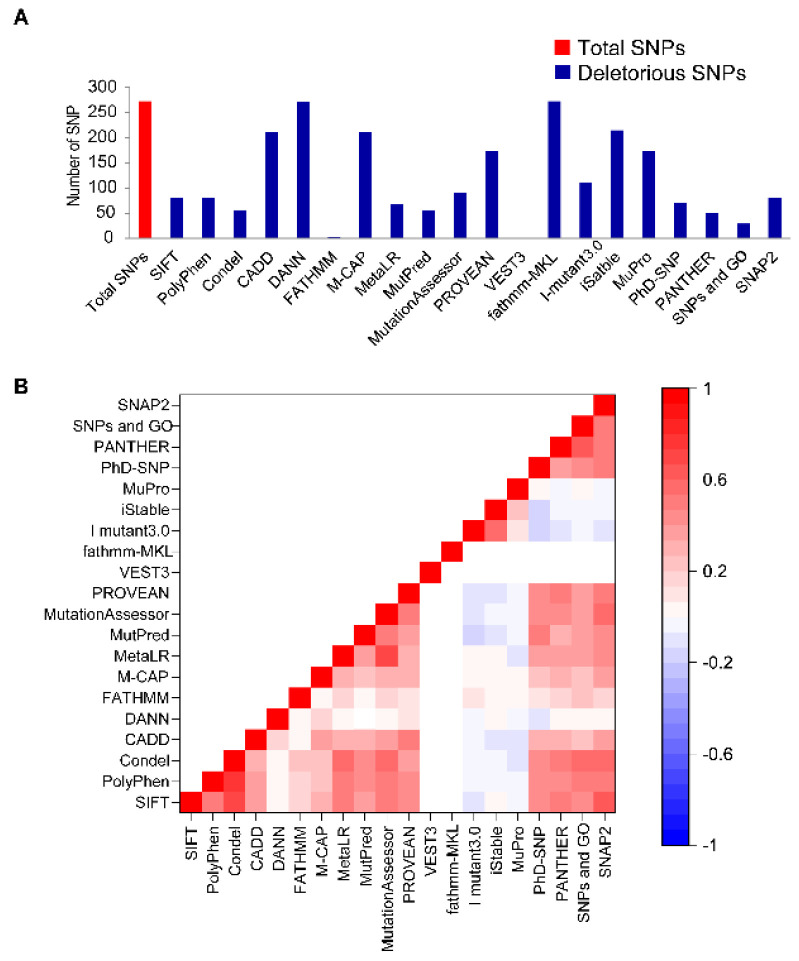
Different computational tools screen and predict deleterious non-synonymous SNPs (nsSNPs) in the *LIS1* gene. (**A**) The bar plot represented a total number of damaging nsSNPs and (**B**) the pairwise correlation expressed by Heatmap illustration color-coded map (red, white, and blue stand for positive, neutral, and negative correlation, respectively) in the *LIS1* gene by various state-of-the-art algorithms.

It has been evidenced that a single amino acid alteration in LIS1 protein can cause variable phenotypic manifestations that could result in diverse lissencephaly phenotypes [37]. Therefore, a total number 273 missense mutations in the *LIS1* gene, which were retrieved from the dbSNP database, have been subjected for risk prediction, using twenty deleterious predicting state-of-art-tools including SIFT, Polyphen, Condel, CADD, DANN, FATHMM, M-CAP, MetaLR, MutPred, MutationAssessor, PROVEAN, VEST3, fathmm-MKL, iMutant3.0, iStable, MuPro, PhD-SNP, PANTHER, SNPs & GO, and SNAP2 (Figure 1). The basis of these algorithms is either sequenced or combined-sequence and structure-based approach (Table S1, Supplementary File S1). The total number of damaging nsSNPs were displayed by Figure 1A (Supplementary File S1), where fathmm -MKL and deleterious annotation of genetic variants using neural networks (DANN) tools were able to determine the majority of deleterious SNPs, accounting for 273 and 271, respectively, and FATHMM predicted the lowest number of SNPs as deleterious, while VEST3 predicted none. The correlation between used algorithms was represented in Figure 1B, where the value of significance was varied by each tool. The majority of the tool showed an almost positive correlation. However, the correlation pattern of four algorithm tools, including FATHMM-MKL, I mutant3.0, iStable, and MuPro with other particular algorithms, was less correlated. Predicted SNPs, those deemed deleterious by the most used algorithms, are usually more likely to be deleterious [33,34]. Therefore, nsSNPs which were considered deleterious by at least eighteen algorithms, were considered high-risk nsSNPs, such as rs121434486, rs587784254, rs587784261, rs587784276, rs121434485, rs757993270, and rs980416636 (Table S2).

**Figure 2:**
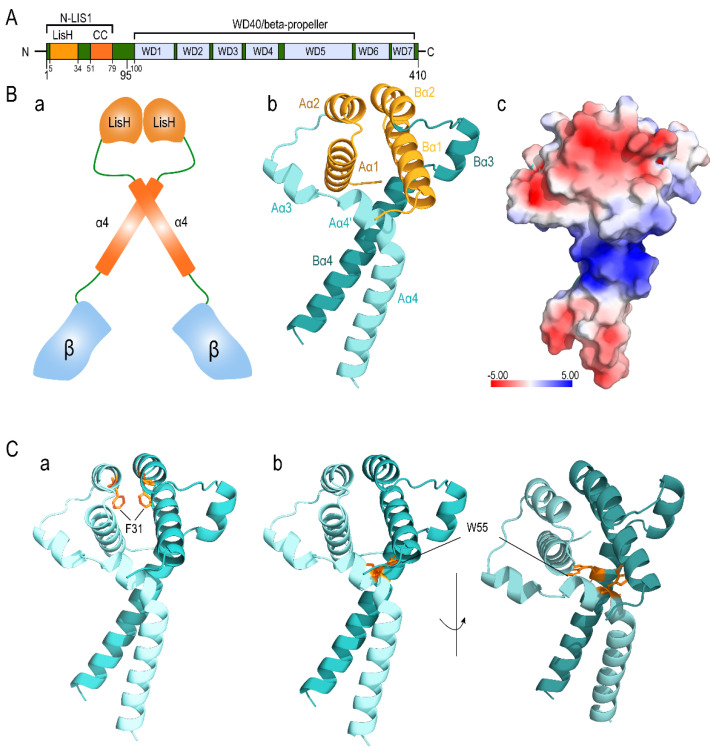
Domain mapping and the position of SNPs in the molecular structure of LIS1. (**A**) Domain mapping highlights the structural feature of LIS1. (**B**) Schematic depiction of LIS1 structure with domains color-coded according to panel A (**a**). Three-dimensional model of the LIS1 N-terminal region highlighting the LisH domain in orange (**b**). The electrostatic surface potential of the LIS1 N-terminal structure was obtained by using the APBS plugin of Pymol 2.5.2. The color scale is represented in kT/e (**c**). (**C**) The detected SNPs in the three-dimensional structure of wild-type LIS1 N-terminal domain are shown by the orange stick for F31 (**a**) and W55 (**b**), respectively.

Structurally, LIS1 confers multiple protein-protein interactions, as it is a member of the tryptophan-aspartic acid (WD) repeat protein family [25]. LIS1 contains seven WD repeats in the N-terminal region [38], while the LisH domain is in the N-terminal region (1–39) [39] and a coiled-coil domain in the middle (40–85) (Figure 2A,B(a)) [34]. The LisH domain is a ubiquitous motif, which serves a general function of LIS1, while the coiled-coil domain is essential for homodimerization. Albeit LIS1 interaction with other proteins is mediated through the WD40 domain, both LisH and coiled-coil domains in the N-terminal is appeared to be essential for various physiological functions of LIS1 (Figure 2B(b,c)) [40]. Deletion of LIS1 N-terminal showed impedance of neuronal migration with an altered neuronal morphology [34]. Interestingly, a common SNP, rs121434486, which caused variation of F31S in the LIS1 protein (Figure 2C(a)) and was reported to be present in a patient with grade 4a (1) lissencephaly with a syndrome of generalized pachygyria and moderate hypoplasia of the cerebellar vermis [37,41,42], is also regarded as the most deleterious by all tools. Furthermore, rs587784254 (W55R) and rs757993270 (W55L) are located in the N-terminal region (Figure 2C(b)), where the residue W55 was reported to be critical for dimerization integrity and allows a dramatic kink that helps to form non-helical structures by downstream residues [43].

Homodimerization of LIS1 is an essential aspect of performing the biological function, mediated mainly through the N-terminal region. Furthermore, Heterozygous animals deleting exon 1 (residues 1–63) exhibit a phenotype consistent with lissencephaly and result in no interaction between the mutant protein and PAFAH catalytic subunits [44]. Therefore, we only considered the variants located in the LIS1 N-terminal region for further systematic analysis by MD simulation, such as rs121434486 (F31S), rs587784254 (W55R), and rs757993270 (W55L), rs121434486 (F31S), rs587784254 (W55R), and rs757993270 (W55L), to check their influence on LIS1 homodimerization and thus loss of function.

### 2.2. Molecular Dynamics (MD) Simulation

All predicted high-risk variants, such as F31S, W55L, and W55R, were subjected to MD simulation along with wild-type. Three separate MD runs of 500 ns (a total of 1.5 µs) for each case were performed. Root mean square deviation (RMSD) analysis was conducted to estimate the equilibrated trajectories, considering the initial structure of the simulation. The RMSD analysis suggests that subsequent stable trajectories were retained within the 200 ns of starting simulation (Figure S1, Supplementary File S2) and maintained stability afterward. Accordingly, trajectories from 220–500 ns (280 ns) from each run of all systems were extracted and concatenated to increase the efficiency of conformational sampling, which were subjected to further analysis. To confirm the sufficiency of the conformational sampling, the cosine content of each sub-trajectory was analyzed and found that all trajectories had a cosine value less than 0.1 (Table S3, Supplementary File S2) [45], indicating that the trajectories were converging and appropriate for detailed analysis [34].

#### 2.2.1. Overall Conformational Changes in LIS1 Variants

The root mean square deviation (RMSD) of Cα atoms of protein was calculated based on the equilibrated trajectories to explain the structural change during the simulations quantitatively. The change of RMSD was identified through comparing with wild-type in both dimer and monomeric forms, which denotes structural similarities [46,47], represented in Figure S2A (Supplementary File S2). Notably, a high mean RMSD value was observed in the variants containing LIS1 structures, where W55R represented the highest mean RMSD in dimeric (Figure S2A(a)) and individual monomers (Figure S2A(b,c)). W55L showed a similar phenomenon. However, F31S showed a reduced mean RMSD of monomer A (Figure S2A(b)) than the wild-type, while the monomer B and dimer increased mean RMSD. In Rg analysis (Figure S2B), both W55L and W55R showed an increased mean Rg value in the dimer (Figure S2B(a)) and monomer A (Figure S2B(b)), whereas F31S showed a reduced Rg than the wild-type. In the case of monomer B, no subtle differences were observed among Rg of wild, F31S, and W55L (Figure S2B(c)). Solvent accessible surface area (SASA) calculation was further considered, which described protein flexibility, suggesting an induction protein flexibility in both dimers (Figure S2C(a)) and monomer B form of W55R (Figure S2C(c)), although it showed low in monomer A (Figure S2C(b)). W55L showed no substantial changes in the overall dimer, despite lowering the SASA of individual monomers. F31S remains unchanged in terms of SASA compared to wild-type. The Rg analysis indicates protein compactness, while SASA denotes the change in the solvent-accessible area, which accounts for protein folding stability [48]. Cumulatively, these analyses, such as RMSD, Rg, and SASA, direct that variants containing structures confer a substantial conformational alteration in the LIS1 structure, which might involve conformational deviations in the nearby areas around the variant location.

#### 2.2.2. Changes in the Backbone Motion

**Figure 3:**
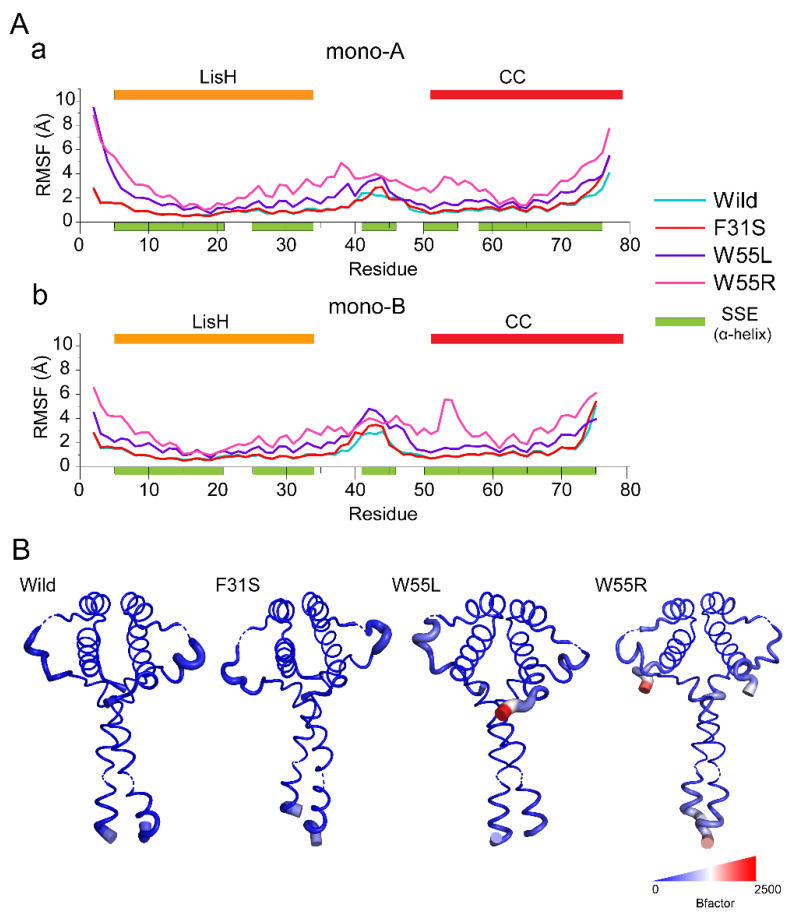
Variant containing structure shows different residual flexibilities during simulation. (**A**) Differences in residual flexibilities are represented by Cα-root mean square fluctuation (RMSF) plot for both monomer A (**a**) and B (**b**). The color bar that is highlighted by specific color reflects helix propensity and domain arrangement as in Figure 2. (**B**) Structural comparison showing the variations as B-factor coloration and thickness, which were calculated from RMSF. The coloring was created using a blue-white-red spectrum with values ranging from 0 to 2500. The thickness of the tube also determines fluctuation, where a more major fluctuation corresponds to a thick tube.

The influence of single amino acid substitution on Cα-RMSF of each residue was assessed using relative residue fluctuations, measured through RMSF (Figure 3), which demonstrates the degree of flexibility induced in LIS1 variants than that of wild-type. Interestingly, as shown in Figure 3A, both variants (W55L and W55R) and wild systems represented asymmetric fluctuation patterns in the RMSF plot (Figure 3B). However, F31S showed an almost similar fluctuation pattern to wild-type, while mild changes were present in the region of 40 to 45 in both monomers (Figure 3A(a,b)). Consistent with RMSD, Rg, and SASA analysis, W55R showed the most noticeable changes in the overall residue among all variants, where a particular jump was seen in the region of 50 to 60 that carries the mutations. Notably, W55 located interfacial region in both monomers, suggesting its precise roles in dimerization and dynamics and flexibility, and thus residual substitution might disrupt the proximal conformation.

#### 2.2.3. Changes in the Conformational Ensembles of the LIS1 Dimer

**Figure 4:**
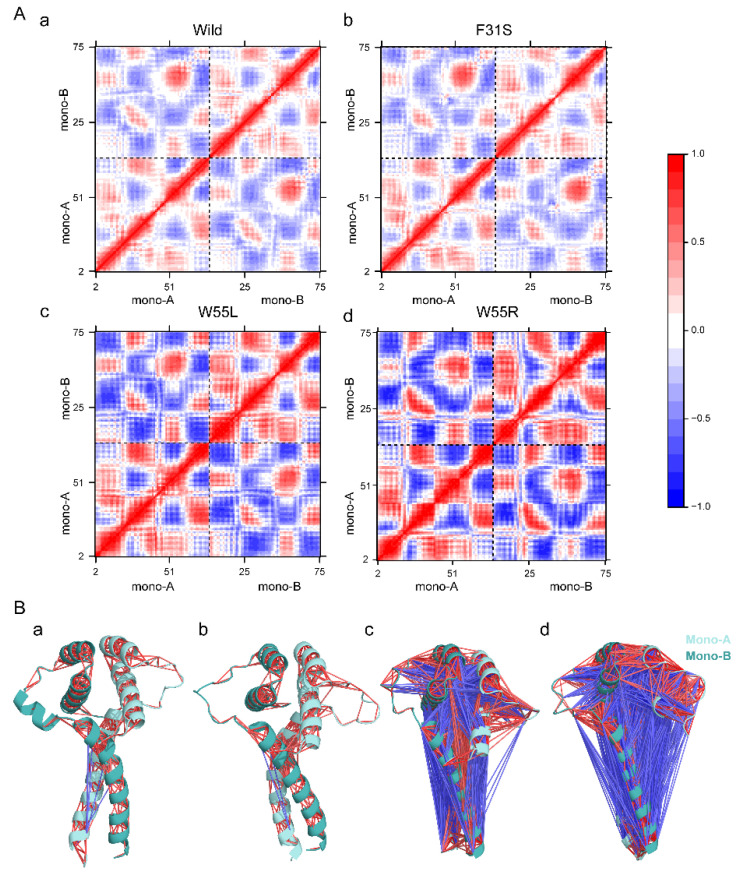
Variants containing LIS1 structures reflect changes in correlated motion. (**A**) The dynamic cross-correlation matrix (DCCM) figure depicts the anticorrelated and correlated movements of each pair of residues in the structure, wild (**a**), F31S (**b**), W55L (**c**), and W55R (**d**). A perfect correlated motion is shown by red (+1), whereas an anticorrelated motion is marked by blue (−1). (**B**) DCCM representation in LIS1 structural view, where lines denote the correlation between two residues. wild (**a**), F31S (**b**), W55L (**c**), and W55R (**d**). A blue line was drawn in anticorrelated movements (−6 to −1), while a red line represents a positive correlation (6 to 1). The intensity of the line color reflects the degree of the association.

The inter-and intra-monomeric correlation motion pattern in all systems was investigated using dynamic cross-correlation matrix (DCCM) analysis (Figure 4). As shown in Figure 4A(a), significant quantities of both correlated and anticorrelated motions were seen to be present in the wild-type. However, these associated movements were induced in high amounts once mutations were incorporated. However, F31S showed a slight decrease in the positively correlated motion and an increase in the negatively correlated movement (Figure 4A(b)), while the dramatic change of correlated motion followed in W55L (Figure 4A(c)) and W55R (Figure 4A(d)), where the degree is higher in W55R than W55L (Figure 4B). An increasing concerted movement in the variants further suggested induced structural flexibility and dynamics, which might weaken the stability of dimerization.

**Figure 5:**
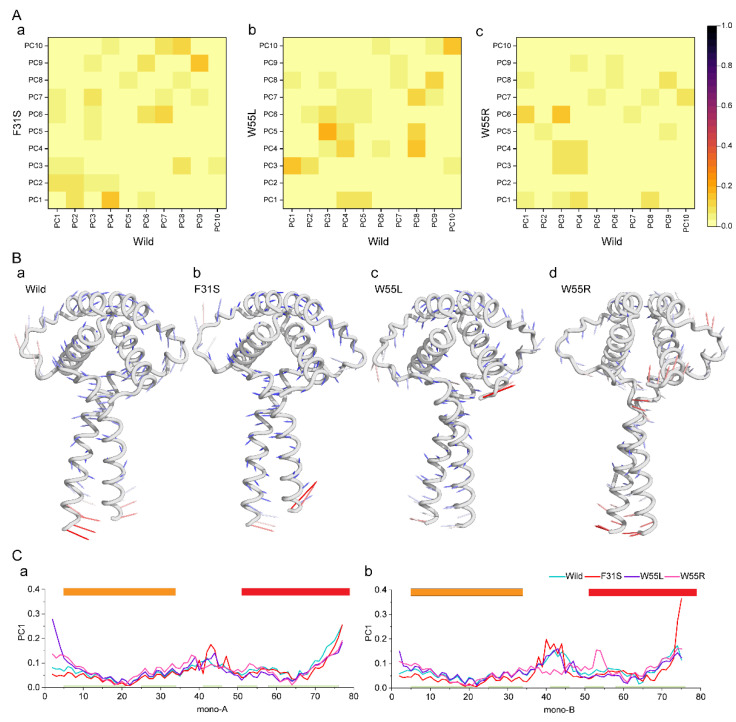
Variations in protein dynamics were revealed by using principal component analysis. (**A**) To demonstrate the similarity and difference among the conformational spaces of wild and variations (F31S (**a**), W55L (**b**), and W55R (**c**)) including structures, the root-mean-square inner product (RMSIP) values of the first PC were counted and plotted as a gradient heat map from yellow to dark red to represent low and high values. (**B**) Porcupine plots were used to show the contributing motions in the first PC for wild (**a**), F31S (**b**), W55L (**c**), and W55R (**d**), respectively. (**C**) Line plot showing the degree of mobility captured in PC1 for monomer A (**a**) and B (**b**).

To characterize the dominating movement by overall conformations, principal component analysis (PCA) was considered, which compresses the ensembles into a sequence of eigenvectors [49,50]. Each eigenvector reflects a section of the protein motion by a phase space characteristic representing protein dynamics [32,34,51]. The essential subspaces that resulted from the PCA of wild and variant ensembles were compared using root mean the square inner product (RMSIP). RMSIP values calculate the similarities and differences between the essential subspaces providing high and low values within a range of 0 to 1 (Figure 5A) [52,53]. The pairwise comparison between the wild and variant structures revealed that variant-containing structures had a different dynamical motion than the wild-type (Figure 5A(a–c)), where significant differences were observed in the first three principal components that cover most of the dynamical motions Figure 5B.

The result was projected onto a 2D plane consisting of the two most typical principal components (PC1 and PC2) to depict the overall conformational landscapes. The introduction of variants induced evident variations in dominant conformational distributions and affected the direction of projected dominant clusters (Figure S3). Interestingly, the F31S variant, which showed a similar fluctuation pattern to wild-type in the previous analyses, demonstrated higher variance than the wild-type, although W55L and W55R represented the highest variance, suggesting increased flexibility and dynamics (Figure S3A–D).

A porcupine plot was created in Figure 5B to display these changes in the protein of dominant motion in the protein structure, and the associated fluctuation is shown as a line plot). This Figure shows that variants containing structure showed a different dynamic motion than the wild-type (Figure 5B(a)). The direction of dominant motion present in the LisH domain of the wild-type was almost consistent with the F31S (Figure 5B(b)). However, the direction of this motion changed in the case of W55L and W55R (Figure 5B(c,d)). A similar pattern was also observed in the case of PC2 (Figure S4). Both W55L and W55R changed the dominant movement in the LisH and CC domains, induced high structural transition open and close motion, whereas W55R induced high mobility in the dimer interface, i.e., the region 50 to 60 (Figure 5C(a,b)), evidencing a possible conformational change in this region.

#### 2.2.4. Changes in the Secondary Structure Organization

**Figure 6:**
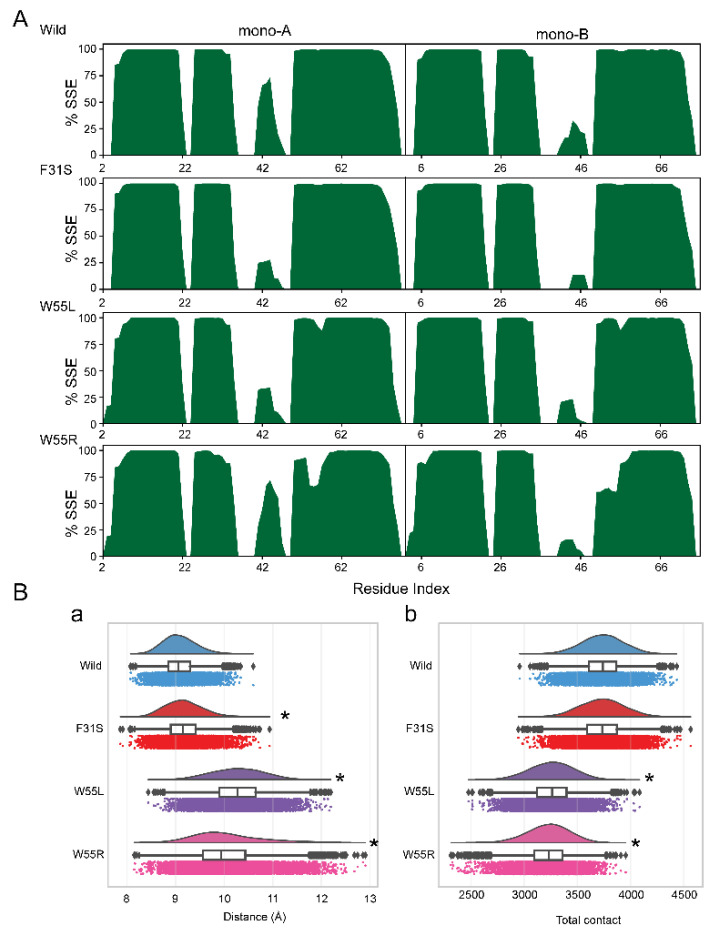
Changes of secondary structural elements and dimer stability. (**A**) The occupancy of the helix structure was estimated as a fraction of a percentage for wild and all variants. (**B**) Raincloud plot [54] shows the distribution and average distance between the centers of mass of monomers (**a**) and total intermonomeric contact (**b**) for all variants and wild-type structures. Annotation *p*-values indicates significant (* *p* < 0.01) in comparison to wild-type.

The total occupancy of essential secondary structures, including helix and strand formation, contributed by each residue in the equilibrated trajectories, had been investigated by the tool called define secondary structure of proteins (DSSP) since RMSF, DCCM, and PCA analyses suggested significant variants induced changes in the LIS1 dynamics. Consistent with the findings from the previous analyses, DSSP showed a reduced secondary structure formation in the residue 50 to 60 in both monomers of the LIS1 dimers (Figure 6A). A significant reduction of helix formation was notable in the monomers A and B in the case of W55R, which occurred near the mutated site. Whereas W55L and F31S reduced helix formation in the region of 40 to 46, suggesting a possible reason for dynamic change and dimer disruption.

#### 2.2.5. Changes in the Stability of Dimers

Since dimerization is the essential aspect for various regulatory functions, mediated through N-terminal fragment, the stability of dimer formation had been assessed in both wild and variants containing structure. The distance between the centers of mass (D_COM_) of two monomers is used to determine the overall tightness of the dimer. Wild-type structure showed D_COM_ averaging 9.081 ± 0.003 Å, while variants have a D_COM_ of 9.168 ± 0.004, 10.27 ± 0.005, and 10.04 ± 0.007, respectively, for F31S, W55L, and W55R (Figure 6B(a)). As seen in Figure 6B(a), the centroid distance between the two monomers is significantly low in wild-type. However, it increased highly due to the presence of variants, where significant variations were noticed in the case of W55L and W55R (Figure 6B(a)).

In addition, total intermonomeric contact formation of LIS1 dimer was analyzed, indicating dimer stability. Figure 6B(b) shows that W55L and W55R reduced the mean total contact between the two dimers (monomer A and B), while no substantial change was between wild-type and F31S. Taken together, it can be concluded that variants reduce dimer stability.

#### 2.2.6. Changes in the Dimerization Potentiality

**Figure 7:**
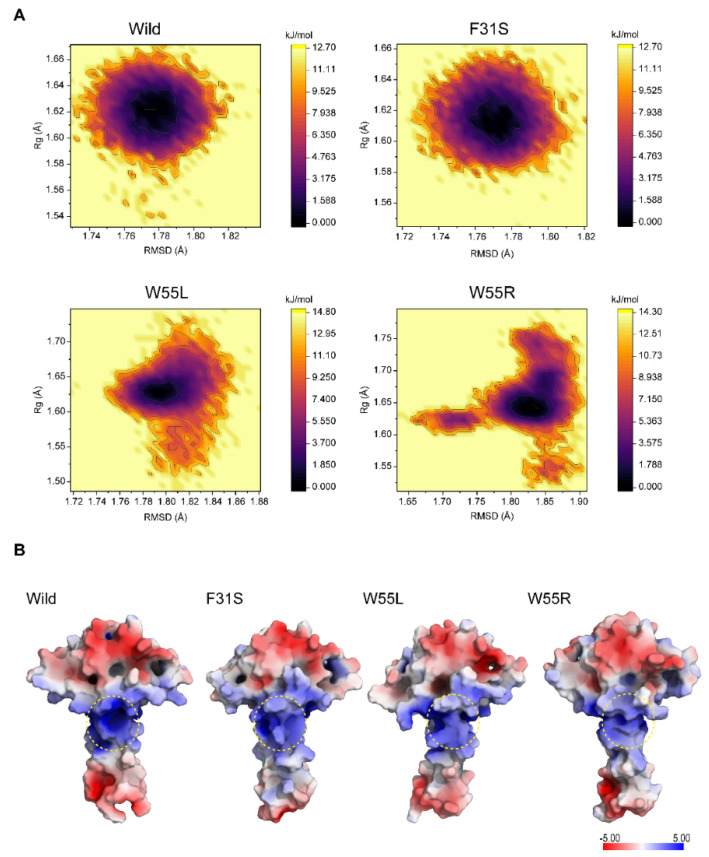
Variants containing structure showed a difference in the positive electrostatic potential in LIS1 structure. (**A**) Free energy landscapes were generated using RMSD and Rg as a reaction coordinate to find energy minimum for wild, F31S, W55L, and W55R, respectively. A color-coded map illustrates the energy state of the protein conformer, with dark purple color indicating a lower energy minimum and yellow indicating a high state. (**B**) The positive electrostatic potential near the dimerization site had been displayed as a surface map for the wild-type and all variants representative structure as determined by the free energy landscape (FEL). A deeper blue zone denotes more electropositive area (>5 kT/e), whereas a red region shows a negative area (−5 kT/e), and a yellow dotted line shows the dimerization site.

The electrostatic potential across the solvated protein surface is critical for macromolecule recognition and binding. Since each monomer of LIS1 confers a positively charged patch of surface-exposed residues in the CC domain, a surface map representing the electrostatic potential was created for all representative structures from variants and wild-type, retrieved from free energy landscape analysis (FEL). FEL provides a more accurate depiction of the protein conformational space in terms of energy and time, distinguishing the kinetic and thermodynamic characteristics of wild and variants containing protein structure during the simulation. In the free energy contour map, the energy minima and energetically preferred protein conformations are shown by dark blue patches, whereas yellow spots represent the unfavorable conformations (Figure 7A). The free energy contour map is shown in Figure 7A shows that the wild-type seems more stable based on its size and the minimum energy region (dark blue area). Both W55L and W55R showed a shallow and narrow energy basin seen during simulation, suggesting low structural stability. Furthermore, both W55L and W55R obtained multiple metastable conformers, suggesting that these variants induced structural transitions different from the wild-type (Figure S5).

The electrostatic potential surface map for each system was created using the lowest energy structure received from the FEL, which also depicts the modest shift of the electrostatic potential in the variants, including structure relative to wild-type (Figure 7A). Although the presence of variants did not demonstrate the widespread loss of positive electrostatic potential, the changes in W55L and W55R were attributed to the exposure of residues ordinarily buried in the wild-type structure (Figure 7B). Whether or not the change of electrostatic potentiality could decrease dimer binding, MM-GBSA binding energy calculations have been performed to estimate the LIS1 dimerization in energetic terms. The MM-GBSA results identified that the most stable conformer under the equilibrium trajectories had the highest binding energy, −158.12 kcal/mol (Table S2). Notably, substitutions destabilized the LIS1 dimer by impairing the monomer-to-monomer dimerization capacity. As a result, F31S, W55L, and W55R showed reduced binding energy of −120.5, −119.37, and −119.45 kcal/mol, respectively (Figure S6).

## 3. Discussion

The current work unveiled three potentially harmful SNPs in the *LIS1* gene using several bioinformatics techniques and verified their deleterious phenotypic consequences using MD simulation. As a result of consensus prediction, three nsSNP, including F31S, W55L, and W55R, were categorized as high-risk SNPs. It is worth noting that single-tool prediction of harmful SNPs is not error-free; hence, various techniques, including both sequence- and structure-based techniques, were used to enhance prediction accuracy [31,55]. Interestingly, these combined efforts successfully categorized F31S as a deleterious SNP, which is popularly known for its association with lissencephaly disorders as well as being routinely used for various biochemical analyses [56].

The current work used microsecond-scale MD simulations to examine the atomic intricacies of the LIS1 dimerization and deduce the mechanism by which the identified variants F31S, W55L, and W55R impair LIS1 dimer formation. During the simulation, a substantial difference was noticed between the conformational change of monomers A and B in all systems, where a high difference was observed in the variant containing structures (Figure S2B). Inter-monomeric interaction in the wild-type structure showed that monomer B contributes interacting residues than monomer A in the dimer formation (Figure S7). Since increased interaction enhances the structure stability, monomer B has a more stable state than monomer A, thus remaining more compact in the simulation.

The result of MD simulation demonstrated that although F31S containing structure showed a degree of fluctuation relative to wild-type structure (Figure 3, Figure 4 and Figure 5), it induces the monomeric conformational change and thus reduces dimer stability (Figure 6 and Figure 7). Interestingly, F31S showed a decreased binding energy in MM-GBSA, which is almost similar to the other variants. Notably, F31S did not produce high dynamics in the LIS1 dimer compared to the other variants, but confers alteration of the secondary structure, increases the interdimer distance, and also changes the electrostatics of the surface, which all together influences dimer binding energy. This observation further explains the mechanism of F31S in the alteration of LIS1 dimer and its pathological severity in lissencephaly. On the other hand, the position W55 is appeared to be critical for dimer formation, which vastly changed both dynamics and stability of LIS1 upon residual substitution, and this finding is also consistent with earlier studies [37]. As evidenced in this study, W55L and W55R both affected the complementarity and electrostatics of the surface, further destroying the original interacting stability at the dimerization interface (Table S4). Overall, the degree of dynamical changes represented by each variant containing structure appears to coincide with disease severity or susceptibility, which might follow F31S < W55L < W55R. Taken together, the dynamics behavior that exists in the wild-type is altered due to the presence of variants. Significantly, variants induced more dynamics and flexibility than the wild-type, which changed the structural composition and increased the intermonomeric distance while reducing the intermonomeric interaction and dimer binding potentiality. Dynamical scenarios, such as those presented in this study, maybe crucial to understanding the implications of LIS1 variants in brain disorders.

## 4. Materials and Methods

### 4.1. Data Retrieval & Deleterious SNP Prediction

For this study, the single nucleotide polymorphism (SNPs) related to *LIS1* gene have been retrieved from the NCBI dbSNP database with respective rsIDs. 270 SNPs have been retrieved and screened using 20 widely accepted tools for predicting the most deleterious SNPs. In silico bioinformatics tools, including SIFT, polyphen, condel, CADD, DANN, FATHMM, M-CAP, MetalR, MutPred, MutationAssessor, PROVEAN, VEST3, fathmm-MKL, I mutant3.0, iSTABLE, MuPro, PhD-SNP, PANTHER, SNPs and GO, and SNAP2 have been associated for this study. SIFT [57] is a PSI-Blast logarithm-based in-silico tool that uses sequence homology and predicts the potential role of amino acid substitution on protein function [58]. The prediction value of SIFT ranges from 0 to 1. Polyphen [59] is a web tool that predicts damaging nsSNPs and their effect on protein structure and function. Polyphen uses the amino acid sequence as input, and it combines information about multiple alignments sequences with structural parameters and homologous proteins to predict an nsSNP and its effect. Condel [60] is a combined method of five prediction tools to identify missense single nucleotide variants. As a result of a combination of five different tools, it could be a better predictor of missense mutations’ impact on the biological activity of proteins. Combined annotation dependent depletion (CADD) is an integrative annotation tool to identify causal variants in genetic analyses. It is built from more than 60 genomic features [61]. DANN [62] uses an artificial deep neural network that scores every possible single nucleotide variant to capture non-linear relationships among features. Functional analysis through hidden Markov models (FATHMM) [63] is software for predicting the functional impacts of coding and noncoding variants. Also, it can distinguish neutral variants from deleterious ones [64]. Clinical pathogenicity classifier M-CAP [65] can identify missense variants with 95% sensitivity. M-CAP is based on a gradient boosting tree classifier model, and it applies 318 features as input into the model. MetaLR uses logistic regression to integrate nine independent variant deleteriousness scores [66]. PROVEAN is a prediction tool based on sequence clustering [67] to predict the modification of protein function due to amino acid substitution [68,69]. MutationAssessor is an online in-silico tool based on evolutionary conservancy [70]. It predicts the functional impact of mutations in proteins [70]. VEST3 is a classifier based on machine learning that can predict human disease involvement with missense variants [71]. fathmm-MKL is a web-based tool that incorporates the functional annotations from ENCODE with nucleotide-based sequence conservation measures to predict the impact of coding and noncoding variants [72]. I mutant3.0 is a computational tool based on a support vector machine that predicts protein stability modification due to single point mutation [73]. PhD-SNP is another tool based on support vector machines that predicts whether a point mutation is a neutral polymorphism or is associated with genetic disorders in humans [74]. PANTHER is a web tool based on evolutionary and phylogenic classification and has become a suitable tool to evaluate lists of genes, and whole genomes, from organisms from the phylogenic tree [75]. SNPs and GO are a framework to predict if a mutation is disease-related or not from the analysis of the protein sequence [76]. SNAP2 is based on a neural network and can predict the effect of the single amino acid mutation in protein function [77]. iStable servers predict the effect of single AA polymorphism on protein stability by integrating three sequence-based tools such as the MUpro, the interpretable decision tree method iPTREE-STAB [78], and I-Mutant2.0 [79]. Whereas MUpro is another tool that can be used to anticipate how protein stability would alter in the presence of nsSNPs, which used both neural networks machine learning and SVM methods [80]. A web-based tool, MutPred uses a random forest algorithm to search for changes in protein structures and dynamics due to variants, as well as predictions about how proteins work, as well as amino acid sequence and evolutionary information [81].

### 4.2. Molecular Dynamics (MD) Simulation

All variants containing LIS1 structure had been created based on the LIS1 N-terminal crystal structure (PDB ID: 1UUJ), which had been downloaded from the protein databank and generated in Schrödinger 2021-2 (Schrödinger, LLC, New York, NY, USA), as previously reported [16,32,82,83,84,85]. Following the structure preparation, Schrödinger 2021-2 (Schrödinger, LLC, NY, USA) was used to incorporate the relevant variants (F31S, W55L, and W55R) into the structure through a mutant residue script, described earlier [31,82,86].

Desmond program of Schrödinger software 2021-2 (Schrödinger, LLC, New York, NY, USA) [87,88] was used for MD simulation to evaluate the conformational changes in proteins dynamic movements with the force field, OPLS4 (Optimized Potentials for Liquid Simulation), as described previously [32,89,90,91,92]. A simulation triclinic periodic boundary box was generated to solvate the structures of wild-type LIS1 and three variants (F31S, W55L, and W55R) where an explicit solvation model (Monte-Carlo equilibrated TIP3P, the transferable intermolecular potential 3 points) was used to submerge each system. The extension of each direction was 10 Å [93]. To control the movement of all covalent bonds, including hydrogen bonds, Lennard-Jones interactions (cut-off = 10 Å) and the SHAKE algorithm [94] were used. The whole system was neutralized using additional counter ions (0.15 M of Na^+^Cl^−^) during the solvation. As mentioned in our previous protocol, the desmond trajectory followed eight stages [92,95]. Concisely, the simulation was started in stage two, followed by Brownian dynamics running under NVT ensemble for 12 ps at 10 K temperature with restraints on solute heavy atoms. In the third stage, the exact temperature of 10 K was maintained in the NVT ensemble for 12 ps with restraints on solute heavy atoms. Subsequently, by following the same parameter with restraint on the heavy solute atom, NPT was used except NVT where 12 ps simulation was performed at 1 bar pressure in the fourth stage. The solvate pocket script was used to solvate the protein cavity at 12 ps in stage five. Restraints on solute heavy atoms were running under NPT ensemble while 12 ps simulation was carried out in stage six, and stage seven was involved in 12 ps simulation without any restraints on solute heavy atoms. Finally, 500 ns simulation was carried out at stage eight following the temperature of 300 K and pressure at 1 bar, and the resulting trajectories were applied for further analysis. Each system was subjected to three separate MD runs, and the trajectory was extracted by choosing a time interval of 100 ps for all simulation trajectories. The Particle Mesh Ewald (PME) was used to determine long-range coulombic interactions, while the RESPA integrator (a motion integration package) [96] was used to regulate all covalent bonds that connected with hydrogen atoms, and the inner time step was 2 fs throughout the simulation. Cut-off 9.0Å was selected for short-range electrostatic interactions, and to analyze the long-range van der Waals (VDW) interactions, a uniform density approximation was chosen for the cut-off value. Nosé–Hoover thermostat [97] with a relaxation time of 1 ps was applied at 300 K temperature and 1 atmosphere pressure. Martyna–Tobias–Klein barostat method [98] with a relaxation time of 2 ps was used to maintain the condition during simulation. After that, the stability of each system was evaluated from the trajectories of MD simulation using RMSD (Root Mean Square Deviation), RMSF (Root Mean Square Fluctuation), and SSE (Secondary Structure Elements) by Schrödinger 2021-2 and Rg (Radius of gyration), H-bond occupancies and SASA (Solvent Accessible Surface Areas) were determined by using VMD software [99]. Consine content of subtrajectories was calculated using the essential dynamics tools of GROMACS [34].

#### 4.2.1. Analysis of Protein Dynamics

The dynamic cross-correlation maps were generated to explain the time-correlated motions in the protein atoms during simulation, which were assessed by Bio3D (an R package software) [100]. The following equation was used to calculate the cross-correlation ratio (*C_ij_*) between the skeleton carbon atoms *i* and *j*.

$$C_{ij=}\langle \Delta_{r_{i}}\cdot \Delta_{r_{j}}\rangle /{\left(\left\langle \Delta r_{i}^{2}\rangle \langle \Delta r_{j}^{2}\right\rangle \right)}^{\frac{1}{2}}$$

Here, the average location of the *i*th and *j*th residues was represented by ∆*r_i_* and ∆*r_j_*, respectively, and the angled brackets “〈〉” indicate the mean time of the whole trajectory. The ranged values to illustrate DCCM were from −1 to +1, where positive values represented a positively-correlated motion, and negative values denoted the negatively-correlated motion.

Further, using principal component analysis (PCA), the flexibility and collective motions of wild-LIS1 and variants [101] were elucidated, where Bio3D packages of R were used to calculate PCA. To obtain PCA eigenvectors values, both covariance matrix of atomic coordinates and eigenvectors were analyzed after removing translational or rotational movements. After that, the orthogonal coordinate transformation matrix carried diagonalization, and the product from this analysis was the diagonal matrix of eigenvalues. Columns were the eigenvectors correlated with the direction of motion relative to the initial coordinates. Eigenvector was connected with eigenvalue and represented the total mean-square displacements (MSD) of the system. The details of the whole system have been discussed mathematically in previous studies [102,103].

#### 4.2.2. Free Energy Landscape (FEL)

A conformational sampling method was used to obtain FEL, which provided all possible macromolecular structural conformations [104]. In FEL analysis, protein stability was defined as a function of the entropy and enthalpy using Gibb’s free energy. FEL of protein was obtained. The following equation has been used in order to calculate FEL:

G_i_ = −K_B_Tln (N_i_/N_max_) (1)

Here, k_B_ indicates Boltzmann’s constant, and G_i_ represents the Gibbs free energy of state, k_B_. Temperature set at 300 k is denoted by the letter T. The population bin I and the most inhabited population bin are determined by N_i_ and N_max_, respectively. The bin without population is assigned as minimum provability by an artificial scale where the energy of different levels was represented using a color-coded model. The lowest energy conformer from FEL was subjected to MM-GBSA calculation using HawkDock web server [105]; the details were described earlier at [106].

### 4.3. Statistical Analysis

SPSS v19 software was used to detect correlations across all used bioinformatics tools. In order to compare the most significant combinations, the *t*-test and single-factor ANOVA tests were utilized for comparison. Student’s *t*-test with a 95% confidence interval was performed to compare MD trajectory data, which were resampled by bootstrapping the R software package to remove autocorrelation [107,108].

## 5. Conclusions

Using a comprehensive bioinformatics approach, the current studies revealed three nsSNPs in the *LIS1* gene, which caused substantial structural modifications in the LIS1 structure, and thus disrupted dimer stability, as shown by MD simulation. Although further experimental validation into the precise function of the regions affected by these variants is needed, this study establishes a solid foundation to understand the molecular basis of LIS1 contribution in lissencephaly such as brain disorders, which can be a solid foundation for further research into targeting the LIS1 with therapeutics as a possible treatment for early brain developmental genetic disorders.

## Data Availability

The data presented in this study are available on request from the corresponding author.

## Supplementary Materials

The following supporting information can be downloaded at: https://www.mdpi.com/article/10.3390/ijms23063109/s1.
