# Supplementary material for: Structural Consequence of Non-Synonymous Single-Nucleotide Variants in the N-Terminal Domain of LIS1
Source: Int J Mol Sci. 2022 Mar 14;23(6):3109. doi: 10.3390/ijms23063109 (PMC8955593; doi:10.3390/ijms23063109)
Supplement: Supplementary file 1 [file ijms-23-03109-s001.zip › Supplimentary File 2.pdf]

Supplementary File 2

Table S1. The prediction cut-off used for the identification of deleterious SNPs in *LIS1* gene.

SL.NO	Name of Tool	Prediction Method	Cut-off Value
1	SIFT	Sequence	≤0.05
2	Polyphen	Sequence and structure	>0.9
3	Condel	Sequence	>0.5
4	CADD	Sequence	>20
5	DANN	Sequence	>0.5
6	FATHMM	Sequence and structure	<–3.0
7	M-CAP	Sequence	>0.025
8	MetaLR	Sequence	>0.5
9	MutPred	Sequence and structure	>0.75
10	MutationAssessor	Sequence and structure	>2
11	PROVEAN	Sequence	≤–2.5
12	VEST3	Sequence	<0.05
13	fathmm-MKL	Sequence	>0.5
14	I-mutant3.0	Sequence and structure	<–0.5
15	iStable	Sequence and Structure	Decrease
16	MuPro	Sequence	<0.0
17	PhD-SNP	Sequence	>0.5
18	PANTHER	Sequence	<– 3
19	SNPs and Go	Sequence and Structure	>0.5
20	SNAP2	Sequence and structure	>5

Table S2. Cumulative deletorious nsSNPs prediction in *LIS1* gene.

rs ID	Substitution	SIFT	PolyPhen	Condel	CADD	DANN	FATHMM	M-CAP	MetaLR	MutPred	MutationAssessor	PROVEAN	VEST3	fathmm-MKL	I-mutant	iStable	MuPro	PhD_SNP	PANTHER	SNPs and Go	SNAP2
rs121434486	F31S	**0**	**0.979**	**0.867**	**29**	**0.998**	−2.56	**0.384**	**0.793**	**0.869**	**2.97**	**−5.48**	0.936	**0.976**	**−1.96**	**Decrease**	**−2.28**	**0.742**	**0.678**	**0.725**	**85**
rs587784254	W55R	**0**	**0.961**	**0.849**	**29.7**	**0.996**	**−3.57**	**0.432**	**0.928**	0.635	**3.86**	**−13.64**	0.795	**0.982**	**−0.87**	**Decrease**	**−1.01**	**0.826**	**0.86**	**0.763**	**80**
rs587784261	V124D	**0**	**0.956**	**0.844**	**29.2**	**0.985**	−0.05	**0.237**	**0.510**	**0.887**	**2.98**	**−5.13**	0.991	**0.985**	**−1.53**	**Decrease**	**−1.65**	**0.862**	**0.662**	**0.699**	**78**
rs587784276	W219C	**0**	**1**	**0.945**	**33**	**0.994**	−1.73	**0.524**	**0.797**	**0.88**	**3.74**	**−12.41**	0.977	**0.998**	**−1.5**	**Decrease**	**−0.46**	**0.96**	**0.959**	**0.698**	**64**
rs121434485	D317H	**0**	**1**	**0.945**	**32**	**0.996**	−2.51	**0.461**	**0.891**	**0.956**	**3.55**	**−6.7**	0.958	**0.994**	**−0.99**	**Decrease**	**−1.69**	**0.922**	**0.841**	**0.638**	**98**
rs757993270	W55L	**0**	**0.949**	**0.838**	**32**	**0.986**	**−3.54**	**0.462**	**0.934**	0.68	**3.86**	**−12.62**	0.615	**0.990**	**−0.74**	**Decrease**	**−0.05**	**0.72**	**0.745**	**0.654**	**72**
rs980416636	G350V	**0**	**0.998**	**0.919**	**31**	**0.998**	−0.21	**0.182**	**0.595**	**0.76**	**2.66**	**−8.9**	0.965	**0.993**	**−0.67**	**Decrease**	**−1.70**	**0.943**	**0.858**	**0.858**	**64**

The bold value in each tool indicate the respective SNP is deleterious, which satisfied the following criterion, SIFT **(≤0.05)**, polyphen **(>0.9)**, Condel **(>0.5)**, CADD **(>20)**, DANN_score **(>0.5)**, FATHMM **(< −3.0)**, M-CAP **(>0.025)**, MetaLR_score **(>0.5)**, MutPred **(>0.75)**, MutationAssessor **(>2)**, PROVEAN **(≤−2.5**), VEST3 **(<0.05)**, fathmm-MKL **(>0.5)**, I-mutant3.0 **(<−0.5)**, iStable **(>0.7),** MuPro **(<0.0)**, PhD-SNP **(<0.5)**, PANTHER **(<− 3)**, SNPs and Go **(>0.5)**, SNAP-2 **(>5).**

Table S3. The cosine content was analyzed for the first four principal components of individual sub-trajectories.

System	PC1	PC2	PC3	PC4
Wild	0.0006	0.4417	0.00145	0.01464
F31S	0.2776	0.1513	0.0013	0.04474
W55L	0.4261	0.3766	0.0369	0.16474
W55R	0.3244	0.2047	0.0271	0.0308

Table S4. Total MM-GBSA binding energy (ΔG_{bind}) of LIS1 dimer.

**System**	**kcal/mol**
Wild	−158.12
F31S	−120.5
W55L	−119.37
W55R	−119.45

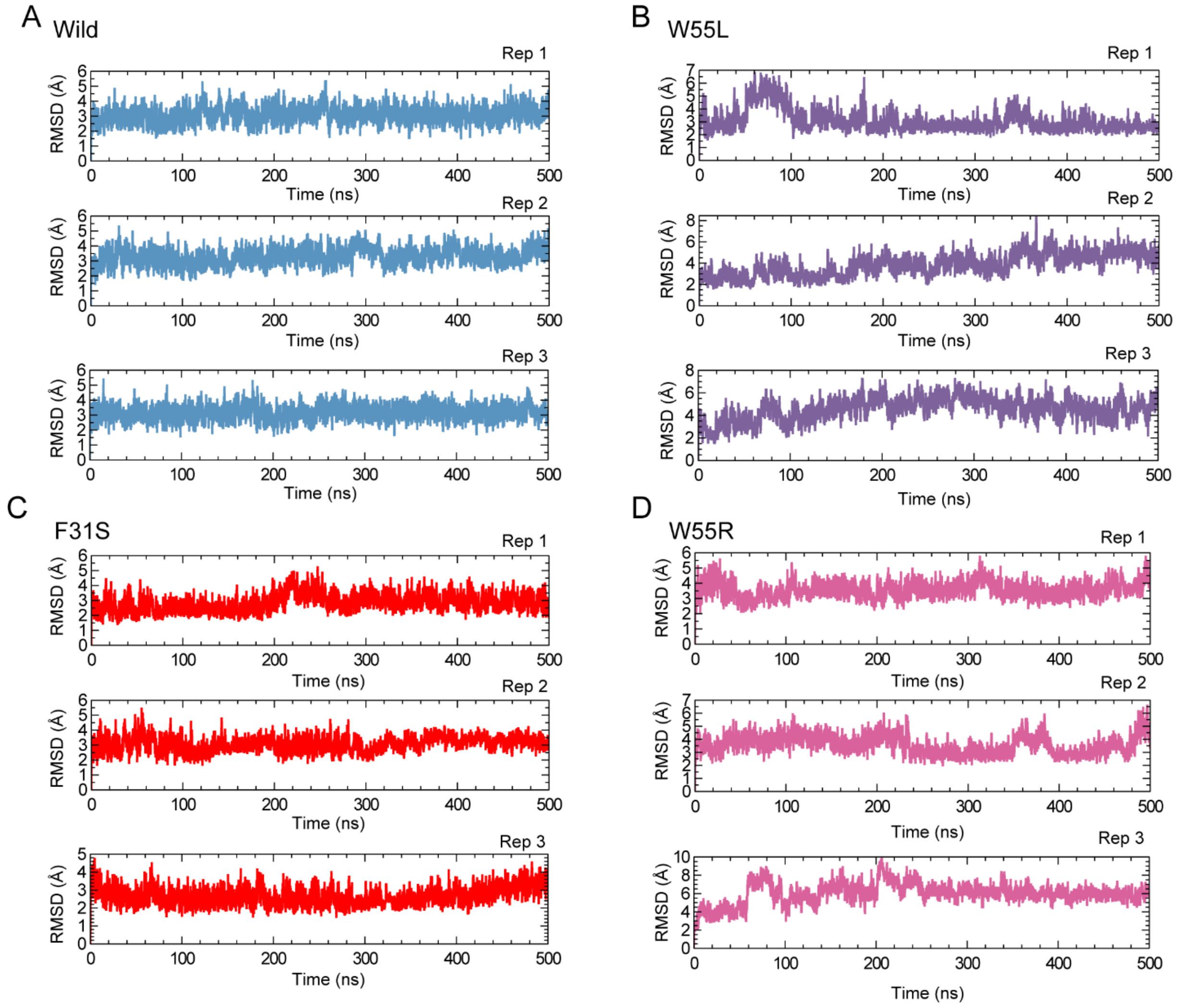

Figure S1. RMSD values generated from each run of the particular system for wild and variant-containing structures were estimated using protein C-alpha of (**A**) wild, (**B**) W55L, (**C**) F31S, and (**D**) W55R, respectively.

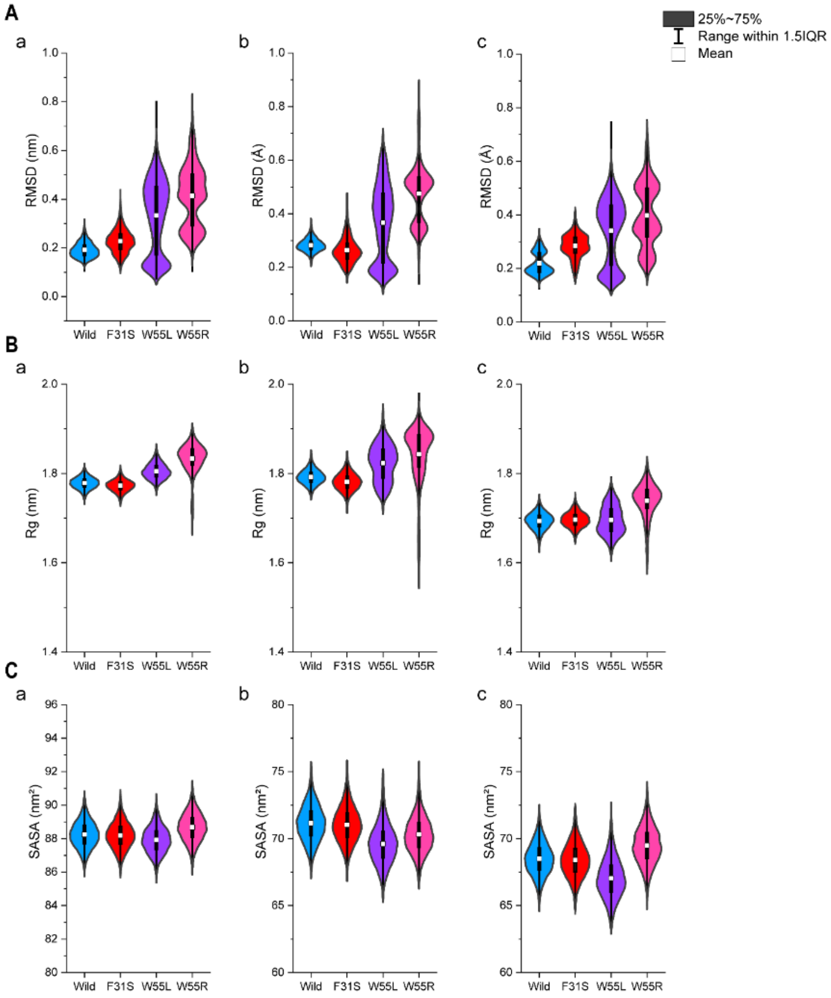

Figure S2. Analysis of conformational stabilities during the simulation. The graphical illustration represents the average difference in the conformational changes between wild and variant-containing structures through RMSD (**A**), Rg (**B**), and SASA (**C**) analyses. In all cases, (**a**) represents the average difference in dimers, (**b**) monomer A and (**c**) monomer B, respectively.

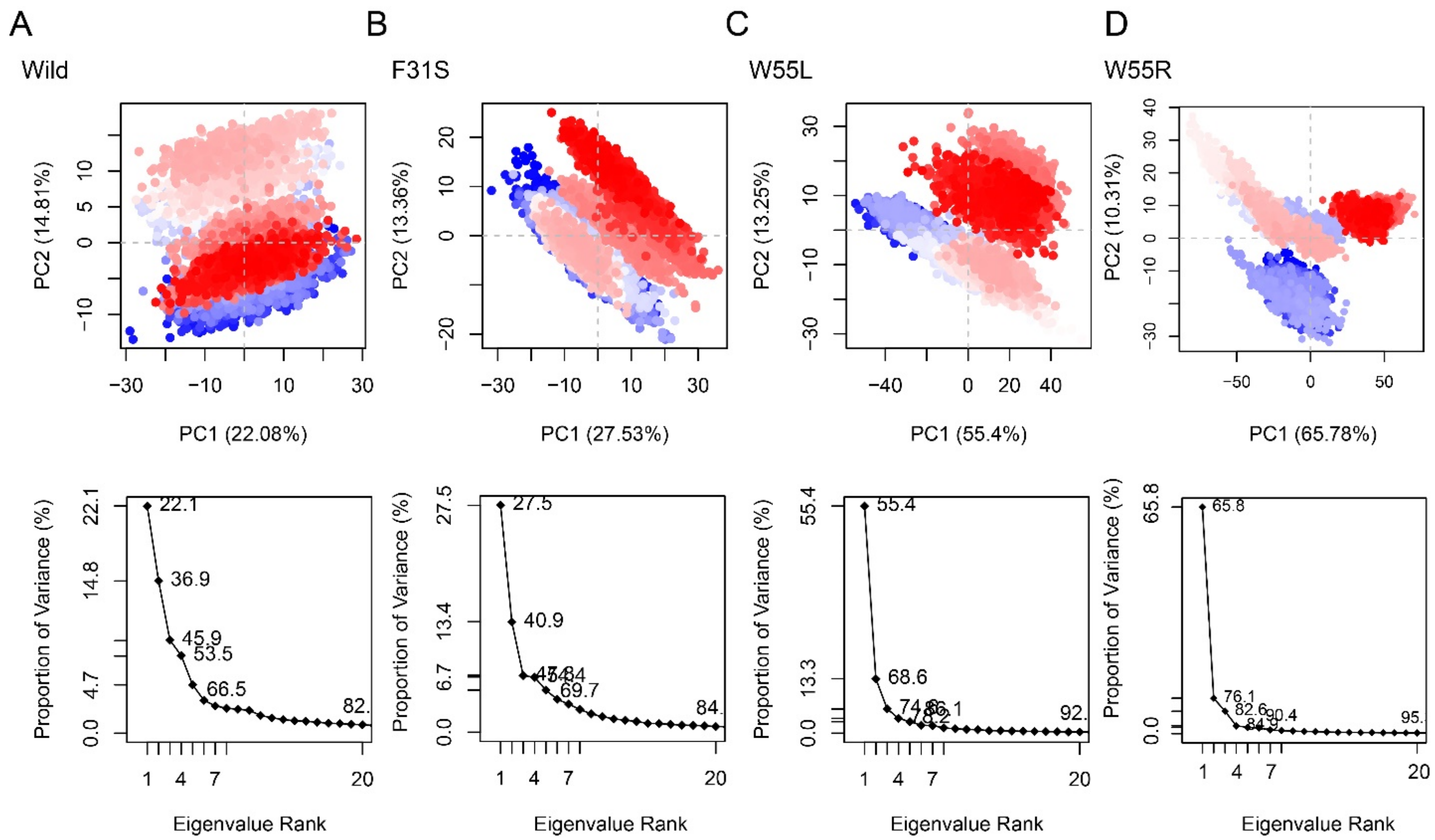

Figure S3. The distribution of trajectory conformers was projected onto principal planes of components 1 and 2, where each dot represents the structure, which was colored by time evolution (From blue to red). The lower plane shows the proportion of variance for all principal components. Here, (**A**) wild, (**B**) F31S, (**C**) W55L, and (**D**) W55R.

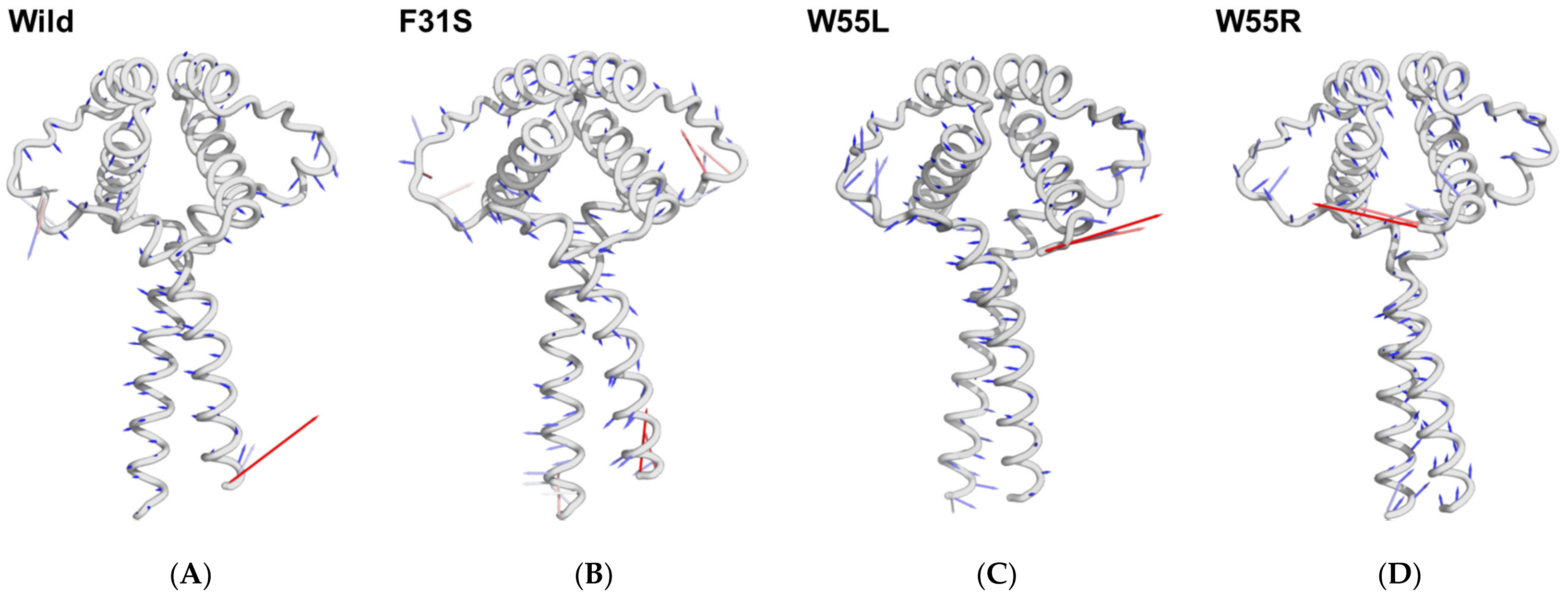

Figure S4. Porcupine plots were used to show the contributing motions in the PC2 for (**A**) wild, (**B**) F31S, (**C**) W55L, and (**D**) W55R, respectively.

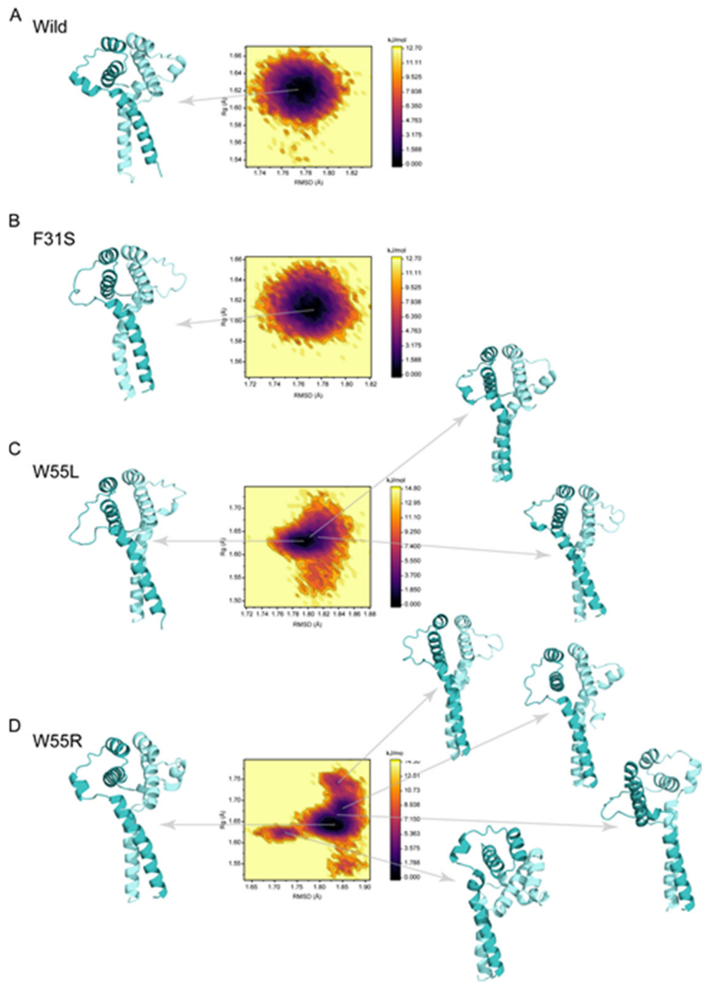

Figure S5. Cartoon representations of the stable and metastable state for the wild and variants, based on the free energy landscape (FEL). Here, A) wild, B) F31S, C) W55L, and D) W55R, which show how variants change the protein conformation. The left side of the figure shows the stable conformer, while the right side represents the metastable state.

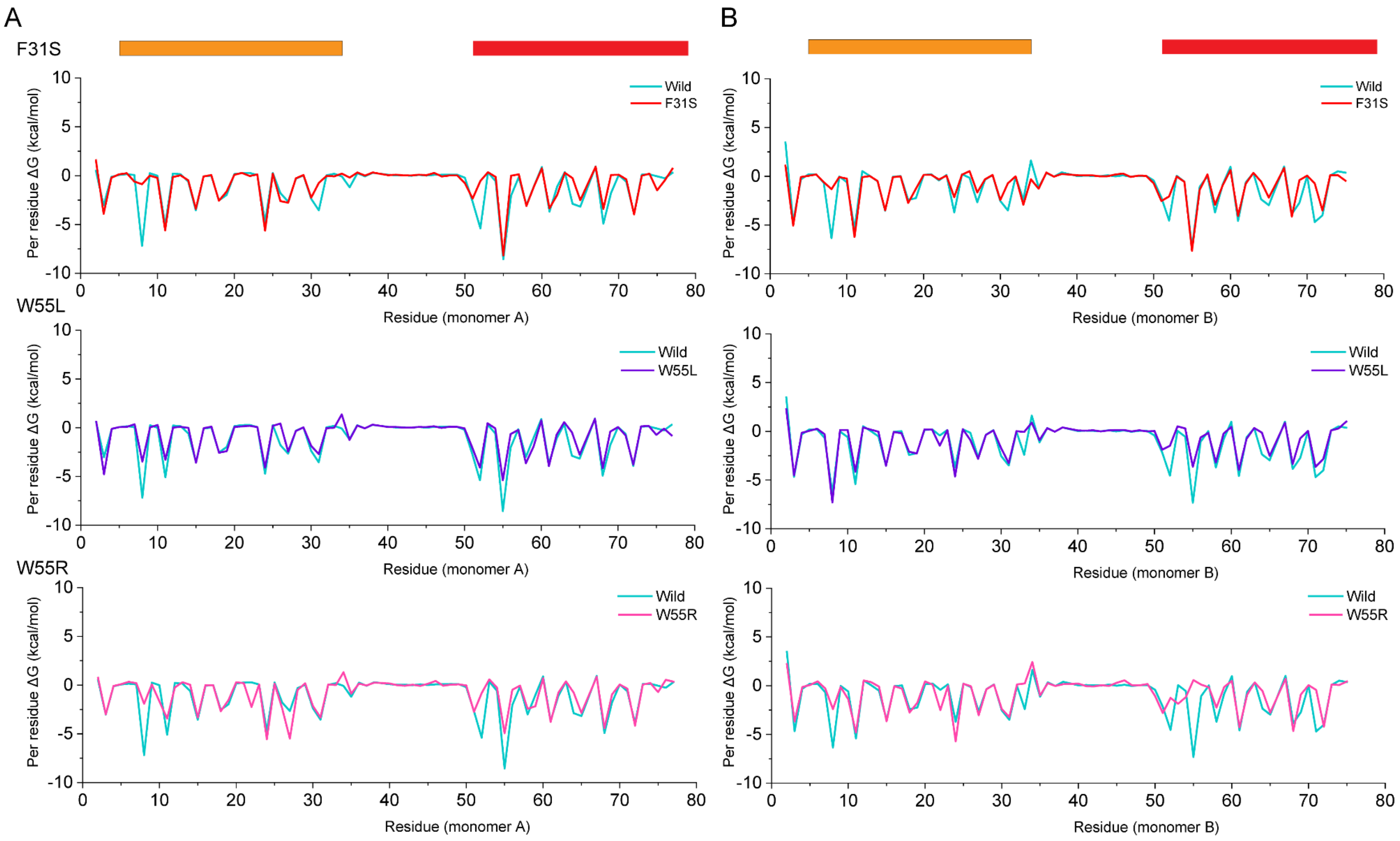

Figure S6. Per-residue decomposition analysis shows the binding contribution by each monomer in LIS1 dimer in the presence of variants, which were compared to wild-type. Here, (**A**) monomer A, while (**B**) monomer B.

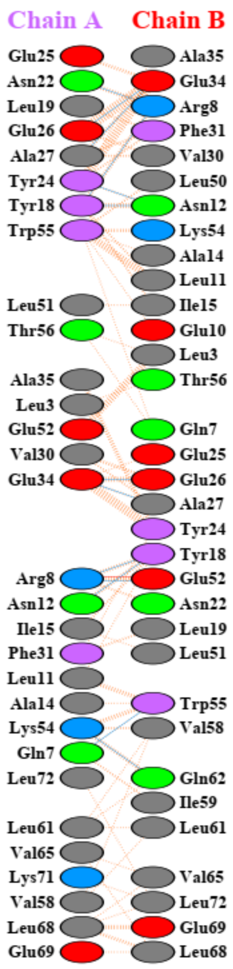

Figure S7. Inter monomeric interaction in the wild-type LIS1 dimer, revealed by PDBsum using PDB, 1UUJ.
